# Follow-Up in Bone Sarcoma Care: A Cross-Sectional European Study

**DOI:** 10.1155/2020/2040347

**Published:** 2020-06-30

**Authors:** Louren M. Goedhart, Andreas Leithner, Joris J. W. Ploegmakers, Paul C. Jutte

**Affiliations:** ^1^Department of Orthopaedic Surgery, University of Groningen, University Medical Center Groningen, Groningen, Netherlands; ^2^Department of Orthopaedics and Trauma, Medical University of Graz, Graz, Austria

## Abstract

**Background:**

Follow-up of high-grade bone sarcoma patients with repeated radiological imaging aims at early detection of recurrent disease or distant metastasis. Repeated radiological imaging does expose (mostly young) patients to ionising radiation. At this point, it is not known whether frequent follow-up increases overall survival. Additionally, frequent follow-up subjects patients and families to psychological stress. This study aims to assess follow-up procedures in terms of frequency and type of imaging modalities in bone tumour centres across Europe for comparison and improvement of knowledge as a first step towards a more uniform approach towards bone sarcoma follow-up.

**Methods:**

Data were obtained through analysis of several follow-up protocols and a digital questionnaire returned by EMSOS members of bone tumour centres all across Europe.

**Results:**

All participating bone tumour centres attained a minimum follow-up period of ten years. National guidelines revealed variations in follow-up intervals and use of repeated imaging with ionising radiation. A local and a chest X-ray were obtained at 47.6% of the responding clinics at every follow-up patient visit.

**Conclusions:**

Variations were seen among European bone sarcoma centres with regards to follow-up intervals and use of repeated imaging. The majority of these expert centres follow existing international guidelines and find them sufficient as basis for a follow-up surveillance programme despite lack of evidence. Future research should aim towards evidence-based follow-up with focus on the effects of follow-up strategies on health outcomes, cost-effectiveness, and individualised follow-up algorithms.

## 1. Introduction

High-grade bone sarcomas are known as rare and aggressive malignancies with chondrosarcoma, osteosarcoma, and Ewing sarcoma as the most common entities [1]. Multimodal treatment including surgery and neoadjuvant therapy by an experienced multidisciplinary team is essential for survival [2, 3]. Disease recurrences, local or metastatic, result in significant reduction of survival [4–9].

Follow-up through outpatient visits with radiological imaging is important to assess postoperative function and to detect local recurrent disease at an early stage. Follow-up is also useful to monitor surgical reconstruction as well as long-term cytotoxic effects of systemic therapy. Osteosarcoma and Ewing sarcoma patients are relatively young though, which results in increased sensitivity to late stochastic effects of ionising radiation due to repeated radiological imaging [10–12]. Repeated follow-up visits raise healthcare expenses and may not lead to improved survival [13].

The most commonly used international guideline for follow-up is the ESMO-PaedCan-EURACAN Clinical Practice Guideline [14]. Based on a recent Asian single-centre randomised study, a less intensive surveillance protocol in terms of frequency and imaging seems noninferior to a more intensive surveillance protocol in terms of survival after treatment of a sarcoma of the limb [15]. An American/Canadian study group has described a large retrospective cohort with data on follow-up frequencies and timing of recurrences, and proposed an alternative (less intensive) follow-up schedule [13]. Based on these findings, more insight into follow-up procedures used in daily practice in Europe is valuable for comparisons. Regional or cultural differences may very well influence decision-making on follow-up procedures.

The European Musculoskeletal Oncology Society (EMSOS) aims to promote advance in science, disseminate knowledge, and promote mutual collaboration for bone sarcoma care between the different affiliated bone tumour centres. This observational cross-sectional study aims to assess follow-up procedures in terms of frequency and imaging modalities in several bone tumour centres all across Europe to compare and improve knowledge as a first step towards a more uniform approach of bone sarcoma follow-up.

## 2. Material and Methods

Data for this observational cross-sectional study were obtained from healthcare professionals. The authors formulated nine questions about organisation of care and produced a digital questionnaire using Google Forms (displayed in Appendix). The questionnaire was not validated. Representatives of EMSOS-affiliated bone tumour centres were approached by the authors based on the EMSOS member archive. We aimed for a proportional distribution across Europe in order to obtain a wide overview. The approached representatives who did return the questionnaire after one digital invitation and one digital reminder were acknowledged and specified as the EMSOS study group. All responses came from orthopaedic surgeons. Observational research among healthcare professionals does not fall under the scope of the Dutch Act on Medical Scientific Research Involving Human Beings (WMO). Data processing was performed using Microsoft Excel 2013 (United States), and analyses were performed using IBM SPSS Statistics for Windows (Version 23.0, United States).

## 3. Results

**Figure 1:**
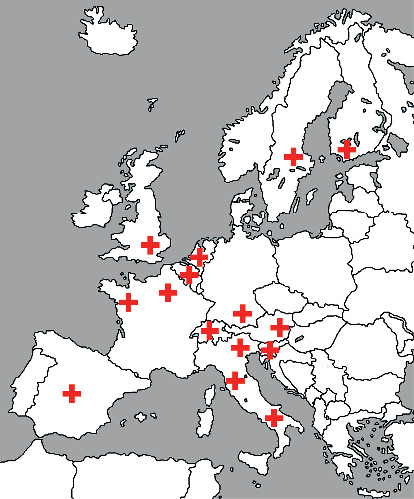
Geographical dispersion across Europe of responding bone tumour centres.

A digital questionnaire was sent to 54 EMSOS member representatives; we received a response of 17 representatives (31.5%) from 12 different countries across Europe. The geographical dispersion across Europe of responding bone tumour centres is displayed in Figure 1.

Using the digital questionnaire as basis, all participating bone tumour centres use a protocol for oncological follow-up after treatment of high-grade bone sarcomas. The bases of these protocols are displayed in Table 1. Authorization for the oncological follow-up protocol was government-based in 33.3% and expertise-based in 66.6% of centres. The guideline as basis for the oncological follow-up protocol used differed across respondents. An international guideline (such as ESMO-PaedCan-EURACAN) was used by 66.6% of centres and a local/national guideline by 33.3%. In terms of duration of oncological follow-up, all participating bone tumour centres attained a minimum of ten years. In two centres (12.5%), the duration of oncological follow-up exceeded ten years. Separate sections and recommendations for osteosarcoma, chondrosarcoma, and Ewing sarcoma were seen in 62.5% of the respondents' follow-up protocol.

Regarding radiological imaging, a local X-ray only was performed during an oncological follow-up visit in one bone sarcoma centre (5.9%). A local and chest X-ray was performed every follow-up visit in eight responding centres (47.6%). In six of the responding centres (35.3%), a local X-ray was performed every follow-up visit with a chest X-ray at a different interval.

We received data points on follow-up intervals from 12 different countries for this study; variations in these intervals are displayed in Table 2. Finland has the shortest follow-up intervals with outpatient visits every two months for the first two years and then outpatient visits every four months up to five years postoperatively. The longest follow-up intervals are seen in the Netherlands, with an outpatient visit every four months between the first and second year of follow-up, downgraded to a follow-up interval of one year between two and five years postoperatively.

Lastly, respondents were asked for their opinion on several topics. Most respondents believe that early detection of a local recurrence as well as of a distant metastasis is important and of clinical relevance for additional treatment. However, some respondents emphasised that survival could depend more on the type and grade of the tumour than on early detection of recurrent disease. Only 25% of respondents believe in added value of an additional, dedicated follow-up guideline for orthopaedic oncology, whereas the vast majority (62.5%) believes that the current international ESMO guideline is sufficient. Differentiation between osteosarcoma, Ewing sarcoma, and chondrosarcoma in a follow-up guideline was found useful by 68.8% of respondents.

## 4. Discussion

The aim of this observational cross-sectional study was to assess follow-up as a first step towards a more uniform approach of bone sarcoma follow-up. This study shows variation in follow-up protocols regarding frequency and use of imaging modalities. With the input from the EMSOS study group, we were able to gather valuable additional information and received several guidelines on oncological follow-up from across Europe.

Limitations for this publication are the observational nature of the study and the disproportional distributed participation from countries and centres. Furthermore, the questionnaire we used was not validated. Despite a digital invitation and reminder, we received a slightly disappointing response rate of 31.5%. This might introduce response bias in this study. On the other hand, we did get a good general impression from most countries which may very well be representative for current policy, and the variation was clearly visible in our data. Regarding the displayed data, the follow-up intervals displayed are based on the input of the study group representatives and arranged by the country. In Germany, there is a known lack of consensus on authorisation of bone sarcoma centres. We, therefore, believe that Germany variability in follow-up intervals and imaging modalities between centres is likely.

As shown in the results, most of the participating bone tumour centres used an international guideline as basis for their national follow-up protocol, and the most commonly used being the ESMO-PaedCan-EURACAN Clinical Practice Guideline [14]. The National Institute for Health and Clinical Excellence (NICE) guideline is an extensive and evidence-based reference as well [16].

In general, follow-up surveillance programmes are based on the implication that early detection of recurrent disease or distant metastasis is of benefit to bone sarcoma patients.

Cool et al. evaluated the efficiency of their local follow-up surveillance programme for extremity bone sarcoma patients in a single-centre retrospective cohort study [17]. Regarding local recurrence, only 38% were detected with follow-up, whereas 62% of patients with a local recurrence presented with symptoms in between a follow-up interval. On the other hand, most pulmonary metastases (64%) were detected using follow-up with repeated imaging while 36% of patients with pulmonary metastases were diagnosed outside the surveillance programme.

The noninferiority trial of Puri et al. outlined that almost 90% of local recurrences are detected by patient themselves, stressing the importance of self-education [15]. Several small retrospective studies have described beneficial results from pulmonary metastasectomy in selected osteosarcoma patients. Beneficial prognostic factors were identified as small pulmonary metastases (<2.0 cm), less than five pulmonary metastases at diagnosis, and a relatively long disease-free interval (DFI) between primary disease and metastatic disease [18–20]. The DFI is an interesting parameter for closer analysis, and Yamamoto et al. associated a DFI <12 months with significantly lower overall survival compared to a DFI >12 months for patients eligible for primary pulmonary metastasectomy [19]. This means that for osteosarcoma patients, recurrence or metastasis within one year of surgical treatment is a negative prognostic factor. This is acknowledged by Cool et al., and their study showed that only 10% of patients with detected pulmonary metastasis survived [17]. In a subsequent study, Cool and Cribb followed 131 high-grade sarcoma patients. Metastatic disease developed in 15 patients, and only 13% was referred for metastectomy. This resulted in a prolonged disease-free survival, but curation was not achieved [21]. A retrospective cohort study from Kim et al. focused on postmetastatic survival. They found that the 5-year postmetastatic survival rate was 31% with a median length of 22 months. Local recurrence prior to metastasis, extrapulmonary metastasis, and poor histological response to preoperative chemotherapy were identified as negative prognostic factors [22]. In summary, the efficiency of follow-up surveillance programmes to detect local recurrences seems to be limited. Furthermore, the effects of intensive follow-up on overall survival remains controversial since early pulmonary metastasis results in inferior prognosis.

Regarding follow-up intervals, earlier detection of local recurrence facilitates the possibility for additional therapy which could lead to a longer subsequent survival period, but will the overall survival be affected? The follow-up interval advised by the ESMO for high-grade bone sarcomas is every 3 months for first two years after start of treatment. After two years, a follow-up interval of 4 months is advised from years 2 to 4. Between 4 and 10 years, a follow-up interval of 6 to 12 months is recommended [14]. For this study, we received follow-up intervals from eleven countries that showed variation. The differences in follow-up intervals and use of repeated imaging as described in the results imply a lack of consensus, which reflects the lack of evidence. None of the responding bone sarcoma centres abide to the follow-up intervals after 2 years as advised in the ESMO guideline. This lack of consensus regarding follow-up intervals among experts for high-grade bone sarcomas is explicated in the 2018 ESMO guideline [14]. Furthermore, the authors of the NICE guideline state that, at the time of publication of their guideline, no comparative studies regarding follow-up strategies and the effects on health outcomes were found [16]. Gerrand et al. acknowledged that evidence is lacking for determination of optimal follow-up intervals [23]. However, Puri et al. found that a less intensive 6-month follow-up interval was noninferior to a 3-month interval in terms of recurrence-free survival and overall survival [15]. Furthermore, a recent retrospective cohort study by Cipriano et al. (including chondrosarcoma, osteosarcoma, and Ewing sarcoma) concluded that most cases of local recurrence occur within the first two years [13]. Late local recurrences (after four years) were uncommon. The highest rates of metastasis were also seen in the first two years for high-grade bone sarcomas with a ratio of 0.66 lung metastases per patient per year. After two years, metastases were seen at lower rates up to ten years. A ratio of 0.018 lung metastases per patient per year was seen 5–10 years after treatment. Based on their study, Cipriano et al. proposed a follow-up protocol for high-grade bone sarcomas. Follow-up should consist of a 3-month interval from 0–2 years, a 6-month interval between 3-4 years, and a 12-month interval from 5–10 years.

Regarding duration of follow-up, ten years was defined as final follow-up moment in 87.5% of the responding centres in this study. In an observational study by Marina et al., adult Ewing sarcoma survivors were compared with their siblings in terms of survival, cause-specific mortality, and chronic conditions [24]. This study with extended follow-up, up to 35 years after treatment, showed that the incidence of late mortality and subsequent neoplasms kept increasing over the years. Chronic cardiac and musculoskeletal conditions related to treatment (chemotherapy, radiation, and surgery) were also seen to increase after 10 years of follow-up. These findings support the need for a lifelong follow-up to assess the late effects of treatment.

In our study, variations were also seen in the use of imaging modalities as well as repeated imaging frequency based on the available guidelines. The ESMO guideline states that imaging of local recurrence or screening for distant metastases could be achieved with local imaging and chest X-ray/CT scanning. Based on the data we obtained, some bone sarcoma patients had up to 10 low-dose chest CT scans in five years while others did not have a single scan. Puri et al. found that even though a CT scan facilitates an earlier diagnosis of pulmonary metastasis, the effects on recurrence-free survival and overall survival are not significantly different compared to a chest X-ray [15]. As mentioned earlier, repeated imaging during follow-up with ionising radiation has proven late stochastic effects in young bone sarcoma patients [10–12].

Several prognostic factors are known for chondrosarcoma, Ewing sarcoma, and high-grade central osteosarcoma. Metastasis at presentation, large primary tumour size, and tumours in the axial skeleton are associated with lower survival for Ewing sarcoma and high-grade central osteosarcoma [4, 5]. For chondrosarcoma, a high-grade tumour and axial localisation of the tumour are poor prognostic factors [6]. Based on these findings, we believe that such prognostic factors could be used to identify high-risk patients after primary treatment. Intensification of imaging during follow-up could be considered for these high-risk patients, despite the lack of evidence whether this will improve overall survival.

We believe that future research should elaborate on the effect of follow-up strategies on survival for comparison with the data presented by Puri et al. and Cipriano et al. [13, 15] Furthermore, cost-effectiveness of bone sarcoma follow-up is an interesting research perspective. Additionally, big data analysis could contribute to the development of an algorithm for individualised follow-up using known prognostic factors. For soft-tissue sarcomas, the PERSARC prediction model is an example to facilitate individualised follow-up [25].

In conclusion, variations were seen among European bone sarcoma centres with regards to follow-up intervals and use of repeated imaging. The majority of these expert centres follow existing international guidelines and find them sufficient as basis for a follow-up surveillance programme despite lack of evidence. Therefore, we believe that future research should aim towards evidence-based follow-up with focus on the effects of follow-up strategies on health outcomes, cost-effectiveness, and individualised follow-up algorithms.

## Figures and Tables

**Table 1:** Baseline characteristics.

	No. of bone sarcoma centres	Authorization basis	Bone tumour guideline	Guideline as basis for oncological follow-up protocol	Minimum follow-up
The Netherlands	4	Government	National	International	10 years
Belgium	5	Expertise	Local	International	10 years
Germany	Not clear^*∗*^	Expertise	National	International	10 years
The United Kingdom	5	Government	National	Local/national	10 years
France	12	Expertise	National	Local	10 years
Spain	10	Expertise	National	International	10 years
Italy	10	Expertise	Local	Local/national	10 years
Sweden	3	Government	National	International	10 years
Finland	4	Government	Local	International	10 years
Austria	4	Expertise	Local	International	10 years
Switzerland	5	Expertise	National	International	>10 years
Slovenia	1	Expertise	National	National	>10 years

**Table 2:** Interval variations in the available follow-up protocols.

	Follow-up interval, 0-1 years	Follow-up interval, 1-2 years	Follow-up interval, 2–4 years	Follow-up interval, 4-5 years	Follow-up interval, 5–10 years
ESMO guideline	2-3 months	2-3 months	3-4 months	6 months	6 months
The Netherlands	3 months	4 months	12 months	12 months	12 months
Belgium	3 months	3 months	6 months	6 months	12 months
Germany	3 months	3 months	6 months	12 months	12 months
The United Kingdom	3 months	3 months	6 months	6 months	12 months
France	4 months	4 months	6 months	6 months	12 months
Spain	3 months	3 months	6 months	6 months	12 months
Italy	3 months	3 months	4 months	6 months	12 months
Sweden	3 months	3 months	6 months	6 months	12 months
Finland	2 months	2 months	4 months	4 months	12 months
Austria	3 months	3 months	3–6 months	6 months	12 months
Switzerland	3 months	3 months	3 months	6 months	12 months
Slovenia	3 months	3 months	6 months	6 months	6 months

## Data Availability

The data used to support the findings of this study may be released upon request to the corresponding author via l.m.goedhart@umcg.nl.

## Appendix

A nine-question digital questionnaire is used on organisation of bone sarcoma care:

(1) Do you use a protocol for oncological follow-up after treatment of high-grade bone sarcomas? (yes, no)
(2) If yes, what is this oncological follow-up protocol based on?
  - Expert opinion/local guideline
  - National tumour guideline
  - International tumour guideline (e.g., ESMO guideline)
(3) According to your protocol, for how many years are bone sarcoma patients (OS, CS, and ES) monitored in terms of oncological follow-up? (5, 10, and >10 years)
(4) Does your protocol contain separate sections/recommendations for OS, CS, or ES? (yes, no)
(5) Which radiological imaging tests are performed during an oncological follow-up visit?
  - Only local X-ray
  - Local X-ray and chest X-ray
  - Local X-ray at every visit and chest X-ray at different interval
(6) In your opinion, how relevant is early detection of a local recurrence of high-grade bone sarcomas?
(7) In your opinion, how relevant is early detection of distant metastases (e.g., lung) from high-grade bone sarcomas?
(8) In your opinion, does a dedicated follow-up guideline for orthopaedic oncology have any added value?
  - No, a local guideline is sufficient
  - No, a national guideline is sufficient
  - No, an international guideline (e.g., ESMO) is sufficient
  - Yes
(9) In your opinion, would a specific follow-up guideline for orthopaedic oncology have to differentiate between OS, CS, or ES? (yes, no).

## Abbreviations

EMSOS: European Musculoskeletal Oncology Society
ESMO: European Society for Medical Oncology
NICE: The National Institute for Health and Clinical Excellence
DFI: Disease-free interval.
